# A Case of Severe Early Childhood Caries Occurring in a Childhood Cancer Patient

**DOI:** 10.3390/children12030261

**Published:** 2025-02-20

**Authors:** Tatsuya Akitomo, Noriko Niizato, Shunya Ikeda, Yuya Ito, Eimi Tabata, Chieko Mitsuhata, Ryota Nomura

**Affiliations:** 1 Department of Pediatric Dentistry, Graduate School of Biomedical and Health Sciences, Hiroshima University, Hiroshima 734-8553, Japan; shunyaikeda@hiroshima-u.ac.jp (S.I.); yuuya@hiroshima-u.ac.jp (Y.I.); amytaba1@hiroshima-u.ac.jp (E.T.); chiekom@hiroshima-u.ac.jp (C.M.); rnomura@hiroshima-u.ac.jp (R.N.); 2 Acacia Kids’ Dental Clinic, Hiroshima 731-0102, Japan; acacia.pedo.dc@gmail.com

**Keywords:** severe early childhood caries, childhood cancer, perioperative oral management, dental anomaly, case report

## Abstract

Background/Objectives: Childhood cancer is the leading cause of death among children, although medical advances are improving the prognosis. During cancer treatment, nausea or vomiting may occur and eating habits may become irregular; therefore, it is important to prevent the development of oral diseases. We encountered a childhood cancer patient with rapidly progressive multiple dental caries, and this report describes the progress. Methods: A boy aged 2 years 9 months was referred for perioperative oral management. No caries were detected in the oral cavity at the initial visit. Results: As the patient had difficulty eating because of nausea and vomiting during cancer treatment, he began to consume probiotic drinks frequently. At 8-month follow-up, dental caries localized to the primary molars was detected. However, caries had occurred in all erupted teeth by 9 months later, confirming the diagnosis of severe early childhood caries. Dental treatment and long-term oral management contributed to good oral health except for dental abnormalities caused by chemotherapy. Conclusions: Childhood cancer patients, particularly at an early age, are at risk of rapid deterioration of oral disease even over a short time period. It is important to cooperate with medical or dental professionals from other hospitals to provide dietary and oral health instruction and continue long-term oral management to improve patients’ quality of life.

## 1. Introduction

Dental caries is one of the most common oral diseases alongside periodontal disease, both being caused by oral bacteria [1,2,3]. These diseases affect the majority of people worldwide, and treatment costs place a significant burden on health services [4]. Although the prevalence of dental caries has decreased significantly over the past few decades, mainly in developed countries, it still affects many people, especially young children and disadvantaged communities [5,6]. Early childhood caries (ECC) is a chronic disease that affects a child’s general state of health, and the American Academy of Pediatric Dentistry defines ECC as the presence of one or more decayed (i.e., having noncavitated or cavitated lesions), missing (because of caries), or filled tooth surfaces in any primary tooth in a child 71 months of age or younger [7,8]. In addition, in children younger than 3 years of age, any sign of smooth-surface caries is indicative of severe early childhood caries (S-ECC). From ages 3 to 5 years, one or more cavitated, missing (because of caries), or filled smooth surfaces in primary maxillary anterior teeth or a decayed, missing, or filled score of ≥4 (age 3), ≥5 (age 4), or ≥6 (age 5) surfaces constitute S-ECC [8].

Although childhood cancer is rare among childhood diseases, it is the leading cause of mortality in children between 1 and 14 years of age [9,10]. According to the 2015 World Health Organization (WHO) report, each year more than 200,000 children are diagnosed with cancer globally, and it is projected that an estimated 21 million people will be diagnosed by 2030 [11,12,13]. Revolutionary advances in cancer diagnosis and treatment have dramatically improved the 5-year survival rate for children with cancer, reaching approximately 80% in developed countries [14,15]. There are currently four treatments for cancer: surgical removal, immunotherapy, radiation therapy, and chemotherapy [16,17]. All interventions carry risks of different complications and side effects, as do the treatments used to manage these adverse events [18]. The side effects of treatment are mainly classified as infectious complications, oral mucositis, nausea and vomiting, or graft-versus-host disease [18]. In addition, the incidence of oral complications during chemotherapy such as ulcers, gingivitis, or xerostomia is higher in children than in adults [19,20,21]. Bonnaure-Mallet et al. investigated oral complications during chemotherapy and reported that tooth brushing significantly reduced the number of affected children [21]. By contrast, in a survey of adults, Kim et al. reported that the frequency and duration of tooth brushing during hospitalization was lower and the use of oral care products decreased in comparison to daily life [22]. Although these reports highlight the importance of oral care during hospitalization, they also suggest a risk of poorer oral hygiene in hospital than in daily life.

Neuroblastoma is the most common extracranial solid tumor in early childhood [23,24]. The prevalence is 7–8 cases per million per year in the UK, around 100 cases per year (8% of childhood malignancy) [25,26]. In Japan, neuroblastoma occurs in approximately 150–200 children each year [27]. Ninety percent of the cases occur in those <5 years of age, with the average age at diagnosis being 2 years [28]. Neuroblastoma originates from neural crest tissue and most commonly manifests on the adrenal glands or thoracic, abdominal, or cervical paraspinal ganglia within the first few years of life, with two-thirds of patients experiencing metastasis to the regional lymph nodes [29,30,31]. The most prevalent locations of metastases are bone marrow and bone, with some cases occurring in mandibular bone [32]. Treatment for patients with neuroblastoma includes a combination of chemotherapy, surgical tumor resection, stem cell transplantation, radiotherapy, and immunotherapy, with the intensity of treatment adapted to risk [31,33]. Therefore, as with other cancers, there is a risk of side effects, and oral management during cancer treatment is important.

We encountered a childhood cancer patient with rapidly progressive multiple dental caries who was diagnosed with S-ECC. This report describes the oral condition and long-term oral management of the patient. Informed consent was obtained from the patient’s guardian to publish this report.

## 2. Detailed Case Description

A boy aged 2 years 9 months was referred from the pediatric department of our hospital with a chief complaint of perioperative oral management. He was born at 28 weeks’ gestation with a height of 35.2 cm and weight of 852 g. Because he was an extremely-low-birth-weight-infant, he was hospitalized in the pediatrics department of our hospital from birth for two months. At the age of 2 years and 8 months, the patient was admitted to the hospital with pain as the chief complaint and was diagnosed with neuroblastoma in the retroperitoneal region and the stage was IV. There was no family history. Chemotherapy, which includes cisplatin, cyclophosphamide, etoposide, and pirarubicin, was performed for six days.

**Figure 1:**
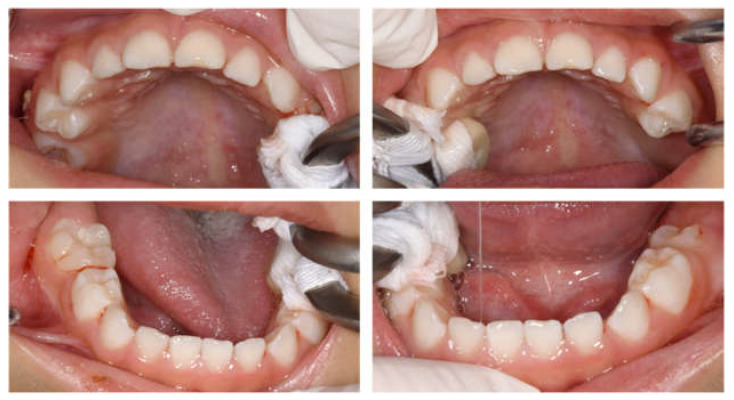
Intraoral photographs at the age of 2 years and 9 months.

Intraoral examinations were performed by a pediatric specialist. At the first visit, 19 primary teeth except the left maxillary second primary molar erupted in the oral cavity (Figure 1). The maxillary left central primary incisor was chipped; however, no dental caries required dental treatment. Three primary second molars had erupted, and the gums were generally swollen and bled easily. The evaluation was performed according to the diagnostic criteria of the Japanese Society of Periodontology, and the patient was diagnosed with plaque-induced gingivitis [34]. He usually had his teeth brushed by his guardian at night, but this had not been possible since being hospitalized. The only drink he took was milk; owing to the effects of cancer treatment, he had difficulty eating meals and did not eat snacks. We conducted oral health instruction or fluoride application and decided to continue with follow-up during hospitalization.

This was followed by four cycles of chemotherapy, and he received autoperipheral blood stem cell transplantation at the age of 3 years and 2 months. Six days after transplantation, mild gingivitis was observed, although no other pathological findings were detected. The guardian had continued to brush his teeth once a day. The left retroperitoneal tumor was removed the next month, followed during the next month by 2 weeks of radiation therapy (19.8 Gy/11 Fr.).

At the dental visit at the age of 3 years 4 months, the progression of dental caries was C2 in the mandibular bilateral primary second molar [35]. As the patient often vomited after eating meals, he frequently drank milk and liquid probiotics. Considering his physical condition, we applied a glass ionomer cement (FUJI IX GP, GC Corporate Center, Tokyo, Japan) filling. We decided to monitor the patient’s progress and continue with dietary advice; however, he was discharged from our institution for treatment at another hospital that month.

**Figure 2:**
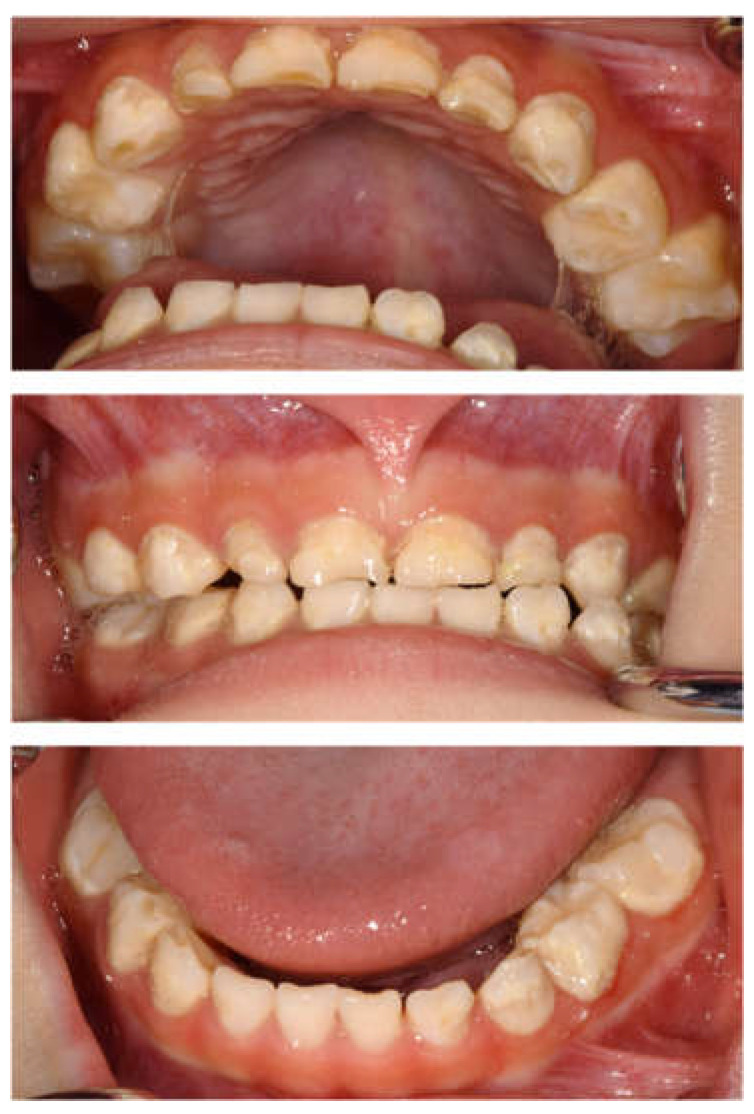
Intraoral photographs at the age of 4 years and 2 months.

**Figure 3:**
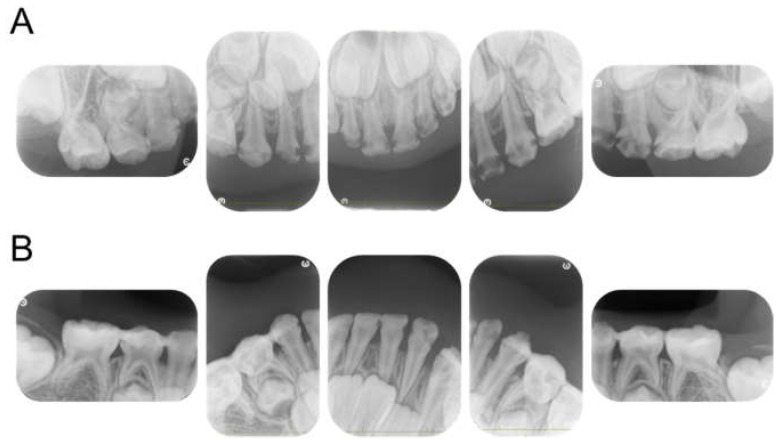
Periapical photographs. (**A**) At 4 years and 2 months. (**B**) At 4 years and 6 months.

The patient returned to our department 9 months later, at the age of 4 years 2 months, for a pediatric consultation. Intraoral photographs revealed multiple severe dental caries (Figure 2). Considering the timing of treatment, radiographic examination was divided into two days (Figure 3A). Radiographic examination of the mandible was carried out 4 months later, and a temporary glass ionomer filling was applied until then (Figure 3B). Two types of glass ionomer cement (FUJI IX GP, GC Corporate Center, Tokyo, Japan, and Vitrebond, 3 M Dental Products, St. Paul, MN, USA) were used. C2 was found in all 20 erupted teeth, confirming the diagnosis of S-ECC. Given the patient’s young age and wider pulp chamber, we chose stepwise removal to avoid pulp exposure [36]. The diagnoses and final restorations are shown in Table 1, and details of the diagnosis of each tooth are given in Figure S1. The mandibular primary central incisors were kept under observation because they represent the site least likely to develop dental caries.

**Figure 4:**
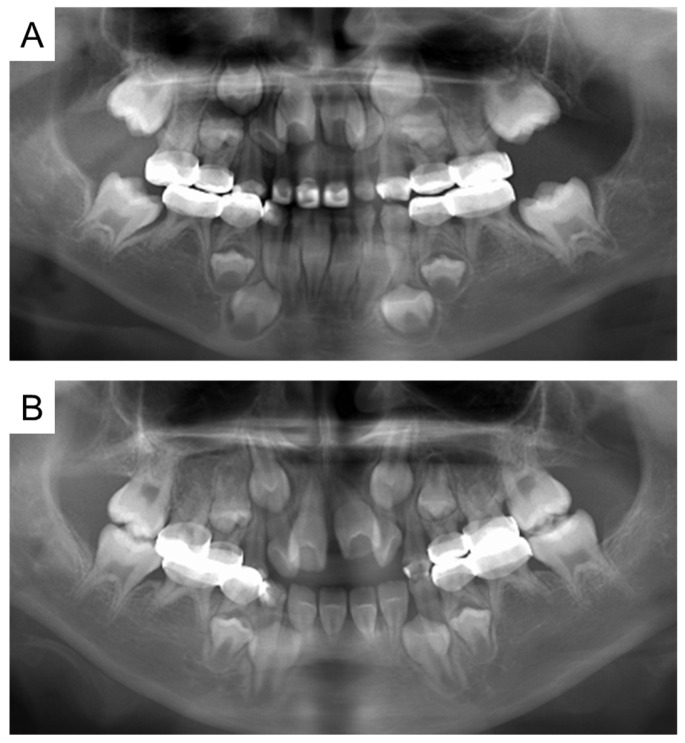
Panoramic examination. (**A**) At 5 years and 11 months. (**B**) At 8 years and 1 month.

**Figure 5:**
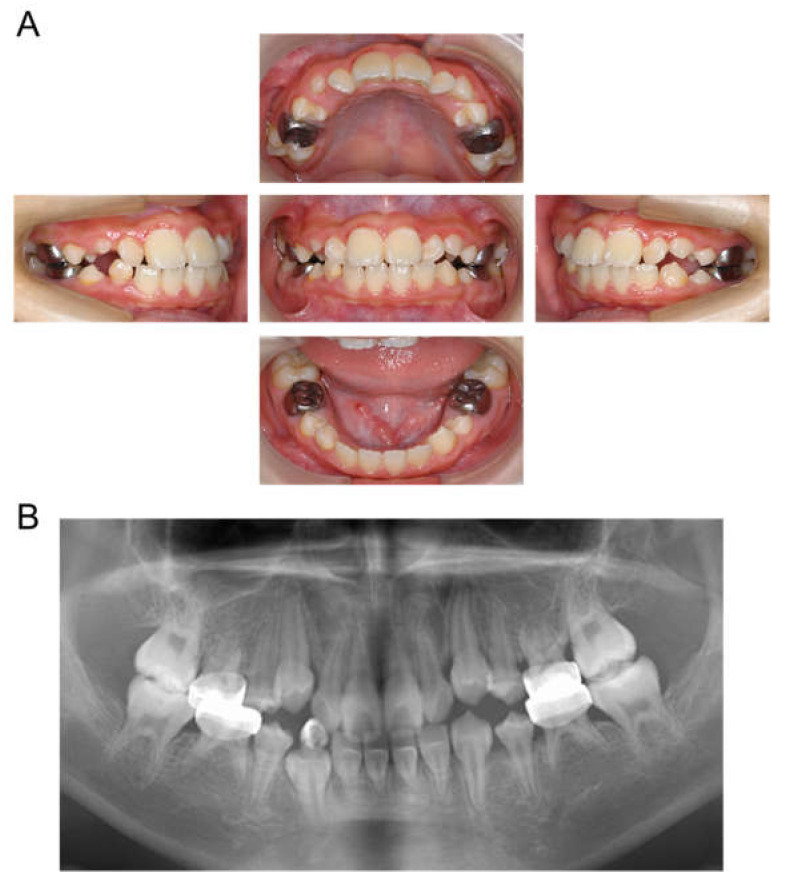
Images at the age of 10 years and 2 months. (**A**) Intraoral photographs. (**B**) Panoramic radiograph.

At the age of 5 years 11 months, the patient’s primary central incisors had mobility. Panoramic examination led to suspected congenital absence of second premolars and second molars; however, there were no dental caries requiring dental treatment. The patient continued to undergo regular checkups and dental treatment as needed. Periodic radiographic examinations confirmed the congenital absence of second premolars and second molars (Figure 4). Short roots were detected in incisors and first molar without mobility and other symptoms, and we continued to observe them. At the age of 10 years 2 months, intraoral examination revealed tooth demineralization and gingivitis, but teeth requiring treatment were not found, and there were no pathological findings on panoramic examination (Figure 5).

**Figure 6:**
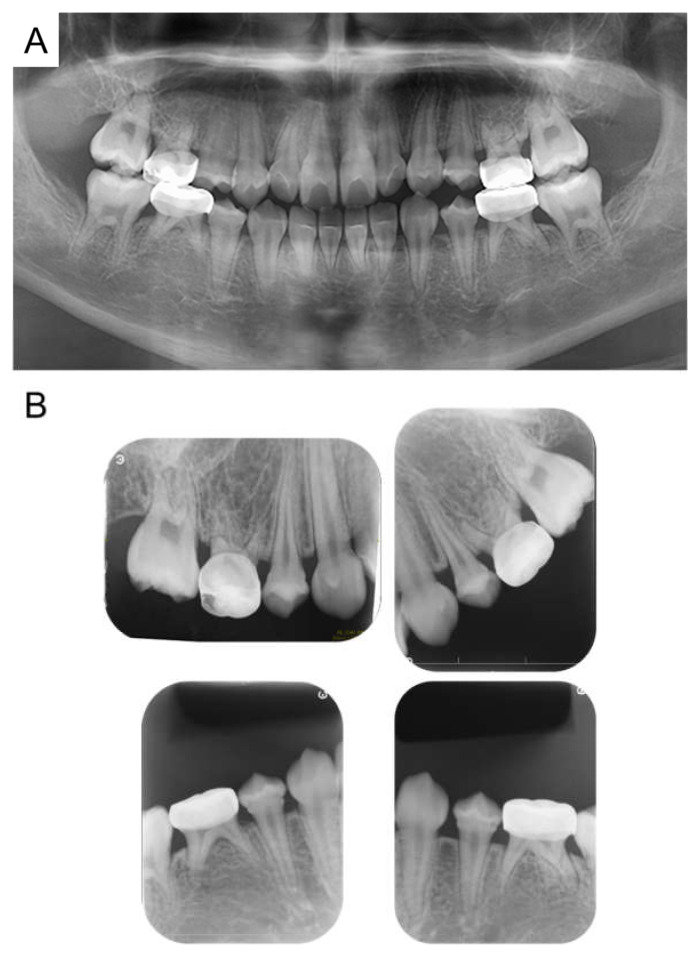
Images at the age of 11 years and 6 months. (**A**) Panoramic radiograph. (**B**) Periapical radiographs.

At age 11 years 6 months, there was no evidence of recurrence of neuroblastoma. All permanent teeth had completed eruption into the oral cavity, and although radiographic examination revealed morphological abnormalities in the first premolar, no teeth showed any pathological symptoms (Figure 6). A hole developed in the preformed stainless-steel crown in the maxillary right primary second molar, which was treated by a replacement. Currently the patient continues to undergo follow-up observation.

## 3. Discussion

Oral function is extremely important not only for chewing and speaking but also psychological well-being [37,38]. Recently, it has been reported that good oral health leads to overall health and well-being, and the importance of oral care has been highlighted [39,40]. It has also been reported that the oral condition of hospitalized patients affects the prognosis of the treatment site and the occurrence of adverse events during hospitalization [41,42]. These results suggest the importance of oral care during hospitalization, particularly by dental professionals. In the present case, perioperative management, including oral care by dental professionals, oral hygiene instruction, and fluoride application, started from the month chemotherapy was administered.

As mentioned above, cancer treatment also causes various side effects in the oral cavity, and oral mucositis is one of the most common adverse effects of radiotherapy and cytotoxic therapy for cancer [43]. Chemotherapy-induced mucositis usually develops within 4–7 days after initiation of treatment and peaks within 2 weeks [43]. On the other hand, radiotherapy has a more gradual clinical course since it is most often administered in small fractions over weeks [43]. In the present case, this patient did not develop oral mucositis during hospitalization, but he had gingivitis and dental caries.

Shayani et al. (2022) reported a significantly greater frequency of gingivitis, and a mean of new caries lesions were observed in children with acute lymphoblastic leukemia who received chemotherapy [44]. Gingivitis has been described as one of the most frequent oral manifestations that affects patients undergoing chemotherapy because of secondary saliva dysfunction and the accumulation of plaque [44,45,46]. On the other hand, dental caries does not occur due to the effect of the chemotherapy itself [44,45,46]. Occurrence of dental caries relates to reduced saliva production, habitual consumption of sugary drinks, or cases of vomiting affect oral hygiene [44]. In addition, during chemotherapy, patients with cancer present a change in oral microbiota with a significant increase in Gram-positive cariogenic bacteria (*Streptococcus mutans* and *Lactobacillus* spp.) [47,48,49].

In the present case, during the 8-month follow-up period, chemotherapy was administered as well as stem cell transplantation, tumor removal, and radiation therapy, while, thanks to oral care and oral hygiene instruction by dental professionals, only mild caries occurred in the primary molars. However, during the 9-month period when the patient did not visit the dentist, the dental caries progressed and he was subsequently diagnosed with S-ECC.

There are two reasons to explain why dental caries progressed so rapidly. First, the patient lived through a period when nausea and vomiting made it difficult for him to eat, and he began drinking liquid probiotics more frequently. Nausea and vomiting are common side effects of many cancer treatments, and there is also often a psychological impact; in particular, nausea is commonly identified as being a distressing aspect of chemotherapy treatment [18,50]. In addition, in children with cancer, malnutrition is common because of tumors, treatment-related factors, and long-term changes in diet, leaving parents concerned about their child’s nutritional status and appropriate food choices for their children [51]. In the present case, the patient had difficulty eating because of vomiting; therefore, although milk was previously given mostly, the frequency of providing prebiotic drinks gradually increased. The etiology of dental erosion is divided into two categories: endogenous, caused by acids entering the oral cavity through reflux or vomiting, and exogenous, caused by environmental factors such as diet, drugs, and lifestyle habits [52,53]. At pH levels below the critical pH for enamel (pH 5.5), tooth minerals are demineralized, and it has been reported that soft drinks with pH values below the critical pH level cause greater loss of surface enamel [53,54]. Recently, promotion of oral health with probiotics has gained considerable attention, and there are some reports on inhibition of *S. mutans*, which is a major pathogen of dental caries [55,56,57]. By contrast, however, Jitpukdeebodintra et al. reported that enamel exposed to drinkable yogurt showed uniform etching and this caused enamel dissolution [58]. The primary dentition is thought to be more susceptible to erosion than the permanent dentition because of its thinner and less mineralized enamel [59]. In addition, Schab et al. pointed out that consumption of milk, dairy products, vegetables, and fruits in children throughout cancer treatment is too low, while the consumption of sugar often exceeds recommendations [60]. Sugar supplementation lowers the pH of the environment, which subsequently increases the number of key bacteria and leads to a shift in microflora, pushing the ecosystem toward demineralization [61,62,63]. Frequent consumption of liquid probiotics may affect the occurrence and progression of dental caries, resulting in S-ECC. Nutritional and dietary guidance during cancer treatment is thus important in the prevention of oral disease.

The other reason is related to the fact that the patient did not visit the dentist during the period when he was receiving treatment at another hospital. Regular dental checkups are important for the early detection of disease [64,65,66]. It is unclear whether the reason for not visiting the dentist was related to a problem with his physical condition or with the medical system, although it is possible that there was a lack of coordination between dental professionals. The arisal of such a situation confirms the importance of cooperation between dental professionals with regard to childhood cancer patients, especially those of a young age.

In recent years, it has been reported that the oral condition in acute ischemic stroke patients at admission is associated with good outcomes and a decreased incidence of hospital-acquired pneumonia, suggesting the importance of oral care for the patient during hospitalization [41]. Regular evaluation for oral mucositis and brushing of the teeth during cancer treatment are common recommendations in several oral care protocols for oral mucositis [67]. In addition, there are also other oral care protocols for the patient during hospitalization [68,69]. In the case of pediatric patients, the American Academy of Pediatric Dentistry strongly recommends that dental professionals assist pediatric cancer patients closely, from diagnosis to follow-up, with the multidisciplinary oncology team [70,71]. On the other hand, Ribeiro et al. (2023) reported that the greatest difficulties of the oral healthcare team were communicating with the medical team and understanding the importance of oral care for the patient’s systemic condition [71]. Oral diseases such as dental caries can progress rapidly during cancer treatment, as in the present case. Dental professionals need to provide proper information and establish close communication among patients, the guardian, and all medical staff involved during hospitalization.

In the present case, pulp preservation was achieved by stepwise removal. In addition, after consultation with a pediatrician, the primary molar region was restored with preformed stainless-steel crowns. A stainless-steel crown protects the dental crown from fracture, reduces the possibility of leakage, and ensures a biological seal [72,73]. During the follow-up period, there were neither tooth fractures nor pulp infection. Patients who have undergone chemotherapy during childhood may develop dental abnormalities, including tooth agenesis, microdontism, and disturbed root development [74,75,76]. Childhood cancer treatment can cause dental abnormalities at an earlier age, the effects of which vary depending on tooth formation [77]. Our patient had tooth agenesis of the second premolar and second molar, microdontia of first premolar, and short roots in the anterior teeth. In childhood cancer patients who have undergone chemotherapy at a young age and in whom no successor permanent teeth have been identified, the importance of restoring the teeth with durable stainless-steel crowns to preserve the dental pulp is evident. Long-term follow-up is important in patients with dental abnormalities [78]. We continue to follow our patient and strive to improve his quality of life.

## 4. Conclusions

In the present case, rapid caries progression was observed in a childhood cancer patient, resulting in a diagnosis of S-ECC. The importance of oral care during hospitalization is well known, but oral diseases can sometimes rapidly become severe. This report provides all healthcare professionals involved in cancer treatment with new risks that pediatric patients face during treatment. With proper treatment and long-term oral management, the patient can live without any oral disease requiring dental treatment. This case highlights the importance of long-term cooperation and intervention between medical and dental professionals during and after hospitalization.

## Figures and Tables

**Table 1:** Diagnosis and final restoration.

Tooth	Diagnosis	Final Restoration
Maxilla		
51	C2	Resin-based composite crown
52	C2	Resin-based composite crown
53	C2	Resin filling
54	C2	Preformed stainless-steel crown
55	C2	Preformed stainless-steel crown
61	C2	Resin-based composite crown
62	C2	Resin-based composite crown
63	C2	Resin filling
64	C2	Preformed stainless-steel crown
65	C2	Preformed stainless-steel crown
Mandible		
71	C2	No treatment
72	C2	Resin filling
73	C2	Resin filling
74	C2	Preformed stainless-steel crown
75	C2	Preformed stainless-steel crown
81	C2	No treatment
82	C2	Resin filling
83	C2	Resin filling
84	C2	Preformed stainless-steel crown
85	C2	Preformed stainless-steel crown

The diagnosis consists of healthy and C1 to C4 [35].

## Data Availability

The original contributions presented in this study are included in the article and Supplementary Materials. Further inquiries can be directed to the corresponding author.

## Supplementary Materials

The following supporting information can be downloaded at: https://www.mdpi.com/article/10.3390/children12030261/s1, Figure S1: The detailing the diagnosis of each tooth.
