# Supplementary material for: A Case of Severe Early Childhood Caries Occurring in a Childhood Cancer Patient
Source: Children (Basel). 2025 Feb 20;12(3):261. doi: 10.3390/children12030261 (PMC11941367; doi:10.3390/children12030261)
Supplement: Supplementary file 1 [file children-12-00261-s001.zip › children-3484232-supplementary.pptx]

## Slide 1
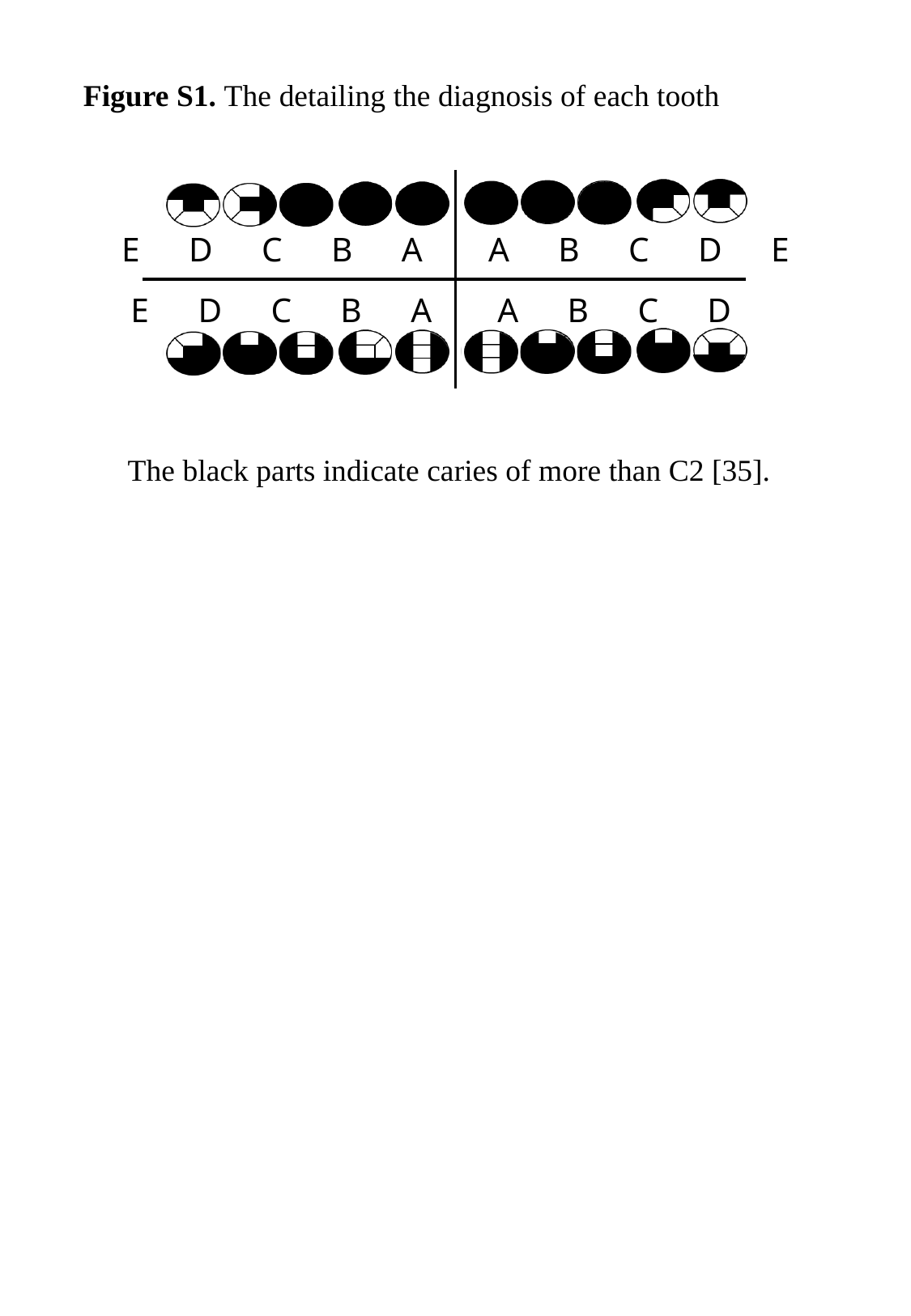

Figure S1. The detailing the diagnosis of each tooth
E　D　C　B　A 　 A　B　C　D　E
E　D　C　B　A 　 A　B　C　D　E
The black parts indicate caries of more than C2 [35].
